# Identification of 2 Novel Species, *Mycobacterium novusgordonae* and *M. shingordonae*

**DOI:** 10.3201/eid3103.240174

**Published:** 2025-03

**Authors:** Kazuki Hashimoto, Yuko Abe, Kiyoharu Fukushima, Yuki Matsumoto, Haruko Saito, Yuri Akamine, Takayuki Niitsu, June Yamauchi, Tadayoshi Nitta, Michio Tanaka, Takuro Nii, Takanori Matsuki, Daisuke Motooka, Kazuyuki Tsujino, Keisuke Miki, Kozo Morimoto, Atsushi Kumanogoh, Shizuo Akira, Shota Nakamura, Hiroshi Kida

**Affiliations:** Osaka University, Osaka, Japan (K. Hashimoto, Y. Abe, K. Fukushima, Y. Matsumoto, Y. Akamine, T. Niitsu, M. Tanaka, D. Motooka, A. Kumanogoh, S. Akira, S. Nakamura); Toneyama Medical Center, Osaka (K. Fukushima, H. Saito, J. Yamauchi, T. Nitta, T. Nii, T. Matsuki, K. Tsujino, K. Miki, H. Kida); Fukujuji Hospital, Tokyo, Japan (K. Morimoto)

**Keywords:** nontuberculous mycobacteria, bacteria, Mycobacterium gordonae, Mycobacterium novusgordonae, Mycobacterium shingordonae, tuberculosis and other mycobacteria, case reports, Japan

## Abstract

We identified 2 novel species, *Mycobacterium novusgordonae* and *M. shingordonae*, from sputum specimens of pulmonary disease patients in Japan. Genetic and biochemical analyses revealed a close relationship with *M. paragordonae*. One *M. shingordonae* case-patient experienced severe progressive infection, highlighting the variation in pathogenicity of the *M. gordonae* clade species.

We report identification of *Mycobacterium novusgordonae* and *M. shingordonae*, 2 novel species in the *M. gordonae* clade, in sputum specimens from 5 patients with chronic respiratory disease in Japan. Written informed consent was obtained from all the participants at enrollment. The ethics board of the National Hospital Organization, Osaka Toneyama Medical Center approved the whole-genome sequencing (WGS) analysis of *Mycobacterium* culture isolates (TNH-R-2020020).

During 2021–2024, staff of Toneyama Medical Center (Osaka, Japan), detected novel mycobacteria strains from 3 patients receiving treatment or follow-up for nontuberculous mycobacterial pulmonary disease caused by other species, 1 patient with a clinical diagnosis of nontuberculous mycobacterial pulmonary disease, and 1 immunocompetent patient with progressive pulmonary infectious disease. Here, we discuss the course of illness and testing for the immunocompetent patient (strain MS1); the other patients are described in the Appendix. 

A 68-year-old woman with a history of chronic gastritis and allergic rhinitis sought care for a chronic productive cough. Chest computed tomography revealed centrilobular nodules and bronchiectasis in the middle and bilateral lower lobes (Appendix Figure 2). Acid-fast bacilli were detected in 61 sputum culture tests. *M. gordonae* was identified 3 times with DNA–DNA hybridization assays (Kyokuto Pharmaceutical Industrial, https://www.kyokutoseiyaku.co.jp), 13 times using AccuProbe (Gen-Probe Inc., https://www.hologic.com), and 1 time using matrix-assisted laser desorption/ionization time-of-flight (MALDI-TOF) mass spectrometry (Bruker Daltonics, https://www.bruker.com). No other pathogenic bacteria were detected. 

The patient initially started clarithromycin monotherapy; her condition progressively worsened. When she was 75 years of age, a left pneumothorax developed that required drainage. At 77 years of age, her providers switched her treatment to erythromycin from clarithromycin because of gastrointestinal intolerance. Despite worsening respiratory symptoms (productive cough, hemoptysis, and dyspnea) and radiologic findings (Appendix Figure 2), she declined the recommended multidrug therapy because of concerns about adverse effects. 

We performed MALDI-TOF mass spectrometry, culture of sputum samples, whole-genome sequencing (WGS), and phylogenetic analysis on all 5 identified strains. MALDI-TOF mass spectrometry identified 2 of the strains as *M. gordonae* and the other 3 strains as unidentified. We processed sputum samples in accordance with the guidance in Clinical Microbiology Procedures Handbook, 5th edition (*1*). After processing, we cultured sputum samples on Ogawa medium at 36°C or in mycobacteria growth indicator tube (MGIT) media at 37°C using a BACTEC MGIT 960 system (BD, https://www.bd.com) (*2*). We subsequently pure-cultured 5 strains on Ogawa medium, analyzed them by WGS, and classified them into 2 groups, MN1/MN2 and MS1/MS2/MS3. Genetic similarity was 99.9%–100% between MN1 and MN2 and 99.1%–100% among MS1, MS2, and MS3. All 5 strains showed an average nucleotide identity (ANI) <95% with the closest known species, *M. paragordonae* (Figure). Phylogenetic analysis showed that the 5 strains and *M. paragordonae* belong to different lineages from *M. gordonae*; however, their mutual relationships are unclear (Appendix Table 1, Figure 1). 

**Figure:**
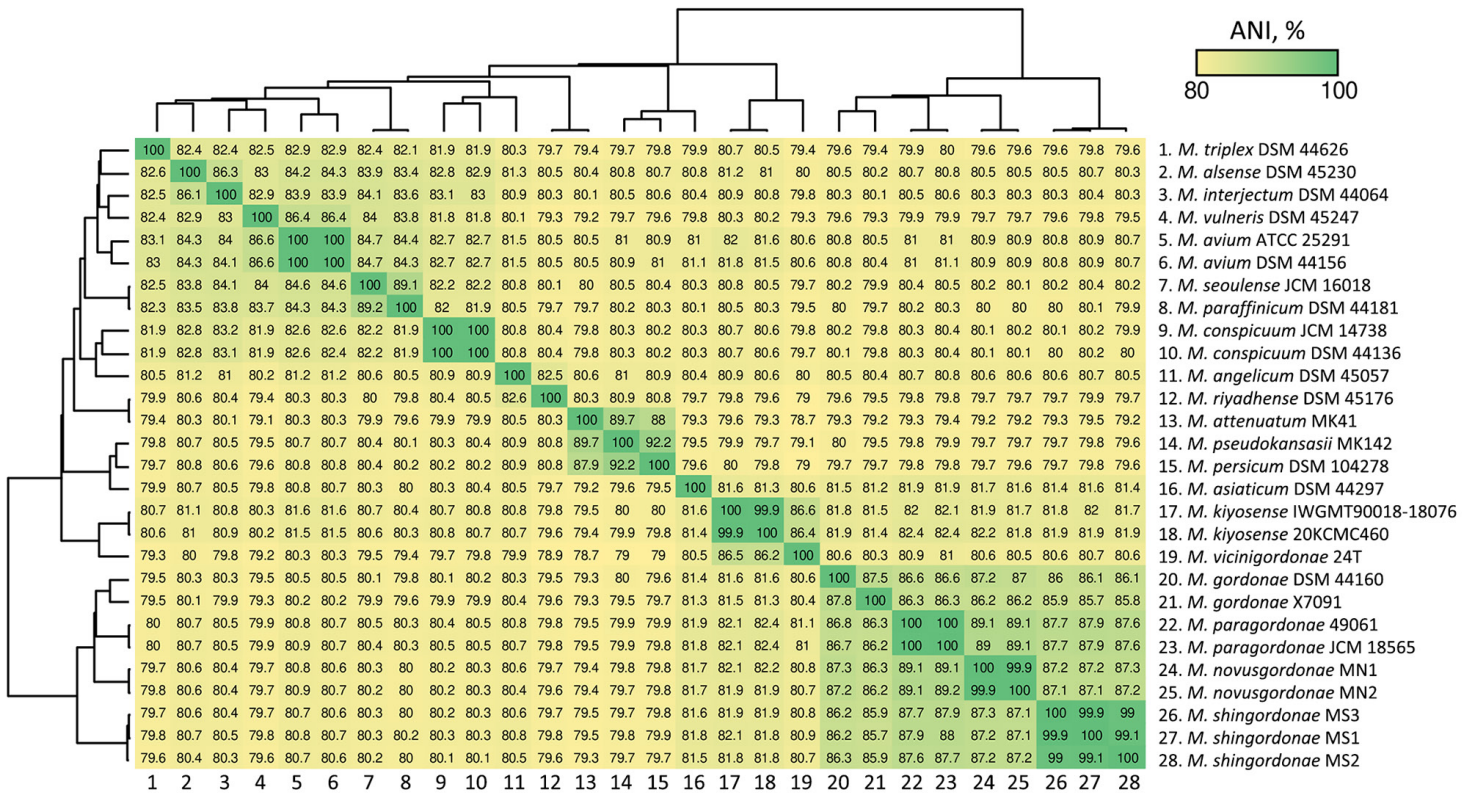
Heatmap showing average nucleotide identity classification of 2 novel mycobacteria species, *Mycobacterium novusgordonae* and *M. shingordonae*, among other *Mycobacteria* species. Heatmap drawn using FastANI (https://github.com/ParBLiSS/FastANI/releases). Species labels in the heatmap were sorted in the same manner as those in the whole-genome sequence-based phylogenetic tree. Numbers at bottom match numbers at right; 24–28 indicate the strains from this study.

WGS confirmed that the MS1 isolates collected at 3 different time points and the MS2 isolates collected at 2 different time points belong to the same species (ANI of MS1, 99.1%–100.0%; of MS2, 100.0%). The new species to which MN1 and MN2 belong was named *M. novusgordonae*, strain type MN1 (TY813, RIMD 1378001, and CIP 11419^T^), and the other new species to which MS1, MS2, and MS3 belong was named *M. shingordonae* strain type MS1 (TY814; RIMD 1379001; CIP 11420^T^) (Appendix). Novus in Latin and shin in Japanese both mean new. 

*M. gordonae* is an environmental acid-fast bacterium traditionally considered to have low virulence and pathogenicity; it primarily causes opportunistic infections in immunocompromised persons (*3*). However, recent studies have identified diverse novel species within this clade, revealing distinct patterns of pathogenicity (*4*–*7*). 

We determined MICs on the basis of recommendations of the Clinical and Laboratory Standards Institute (*8*). Of note, the MN1–2 and MS2–3 strains exhibit low MICs for clarithromycin, ethambutol, and rifampin, whereas the MS1 strain showed high MIC for clarithromycin, in parallel with the detection of an A2058G mutation in the *rrl* gene. We performed biochemical tests on 4 strains: MN1 (*M. novusgordonae* type strain), MS1 (*M. shingordonae* type strain), *M. paragordonae* (RIMD 1369002, CIP 112418), and *M. gordonae* (ATCC 14470^T^) (Table; Appendix Table 3). The MN1 and MS1 strains were nearly identical; they had similar characteristics as *M. paragordonae* but were distinct in their ability to grow at 37°C. MN1 and MS1 isolates and *M. paragordonae* were positive for nitrate reduction. All strains showed positive reactions for catalase, 3-day arylsulfatase, and telluric acid reduction tests.

**Table:** Biochemical assay results for 4 strains of *Mycobacterium* spp. in study identifying 2 novel species*

**Characteristic**	***M. novusgordonae* (MN1)**	***M. shingordonae* (MS1)**	***M. paragordonae* (CIP112418)**	***M. gordonae* (ATCC 14470)**
**Growth at 37°C**	+	+	−	+
**Growth detectable after 7 d**	+	+	+w	+
**Catalase**	+	+	+	+
**Urease**	−	−	−	−
**Nitrate reduction**	+	+	+	−
**3-d arylsulfatase**	+w	+	+	+
**Telluric acid reduction**	+	+	+	+
**Colony color**	Yellow	Yellow	Yellow	Orange
**Pigmentation**	Scoto	Scoto	Scoto	Scoto
**Colony morphology**	Smooth	Smooth	Smooth	Smooth

*Schoto, scotochromogenic; w, weak; +, positive; −, negative.

In conclusion, we identified 2 novel mycobacteria species within the *M. gordonae* clade that are more closely related to *M. paragordonae* than to *M. gordonae*. One patient experienced a progressive infection, revealing the pathogenicity of this novel strain and diversity within the *M. gordonae* clade.

Appendix Additional information about identification of *Mycobacterium novusgordonae* and *M. shingordonae.*
