# Supplementary material for: Identification of 2 Novel Species, Mycobacterium novusgordonae and M. shingordonae
Source: Emerg Infect Dis. 2025 Mar;31(3):624–7. doi: 10.3201/eid3103.240174 (PMC11878325; doi:10.3201/eid3103.240174)
Supplement: Appendix — Additional information about identification of Mycobacterium novusgordonae and M. shingordonae. [file 24-0174-Techapp-s1.pdf]

Article DOI: https://doi.org/10.3201/eid3103.240174

EID cannot ensure accessibility for supplementary materials supplied by authors. Readers who have difficulty accessing supplementary content should contact the authors for assistance.

Identification of 2 Novel Species, *Mycobacterium novusgordonae* and *M. shingordonae*

Appendix

Case Descriptions

Case MN1

A 61-year-old female patient with familial hypercholesterolemia presented with a 1-month history of cough and hemoptysis. She was diagnosed with *Mycobacterium avium* pulmonary disease after multiple sputum cultures tests. She was treated with rifampin, ethambutol, and clarithromycin, which resolved the hemoptysis and achieved negative culture conversion. At 62 years old, an unidentified mycobacterium was detected by matrix-assisted laser desorption/ionization time-of-flight mass spectrometry (MALDI-TOF-MS) in a sputum acid-fast bacilli culture test.

Case MN2

A 65-year-old male with a history of pulmonary tuberculosis successfully treated with multi-drug therapy at the age of 60 resented with hemoptysis and was diagnosed with simple pulmonary aspergilloma, for which he received itraconazole. Treatment was discontinued after stabilization, and the patient remained clinically stable for several years. Acid-fast bacilli

cultures were consistently negative for a long time. However, at 72 years old, *Mycobacterium gordonae* was once detected using MALDI-TOF-MS. Chest computed tomography (CT) revealed bilateral apical cavities, scattered nodular shadows, and bronchiectasis; however, there was no significant progression over time, and his symptoms were mild and stable. At 73 years old, he was transferred to another hospital.

Case MS2

A 57-year-old male patient with no notable medical history, initially diagnosed with *M. avium* pulmonary disease during a routine health check, presented with an abnormal chest radiograph. He was treated with rifampin, ethambutol, and clarithromycin; however, disease progression with hemoptysis and worsening CT findings led to lingular segment resection. Postoperatively, sputum cultures were negative and antibiotics were discontinued.

At 69 years old, he developed a productive cough. Chest radiography and CT revealed atelectasis in the right upper lobe. *M. avium* and *Mycobacterium abscessus* subsp. *massiliense* were detected once. Subsequently, at 70 years old, an unidentified mycobacterium was detected by MALDI-TOF-MS in a sputum acid-fast bacilli culture test. Chronic airway infection was thought to have obstructed and narrowed the bronchi postoperatively. Airway clearance therapy with erythromycin and expectorants was initiated, which improved the atelectasis. Since then, no acid-fast bacilli have been detected and the patient's condition has remained stable.

Case MS3

A 76-year-old man with a history of peptic ulcer, glaucoma, and hypertension was referred to our hospital. Chest radiography revealed an abnormal chest shadow. CT showed a calcified nodule, infiltrates, and linear opacities in the middle lobe. Sputum tests for acid-fast bacilli and anti-GPL-core IgA antibody were negative. Considering the progressive enlargement

of the nodule, bronchoscopy was performed at 77 years old; however, no malignancy or mycobacterial infection was detected. The patient was clinically diagnosed with non-tuberculous mycobacterial pulmonary disease and erythromycin therapy was initiated, resulting in improvement of the radiographic findings. At 79 years old, an unidentified mycobacterial species was detected in the sputum cultures. The patient remained stable and no treatment was administered.

Methods

WGS Analysis

WGS analysis was performed using MinION (Oxford Nanopore Technologies, Oxford, UK) and MGISEQ-2000RS (MGI Tech, Shenzhen, People's Republic of China). Genome assembly for the MN1 and MS1–2 strains was performed using flye 2.9 (*1*) for long reads obtained from MinION, and sequencing errors were corrected with pilon 1.22 using short reads obtained from MGISEQ-2000RS. For MN2 and MS3, WGS analysis was performed using MGISEQ-2000RS (MGI Tech, Shenzhen, People's Republic of China), and genome assemblies were obtained using Unicycler v.0.5.0 (*2*).

Calculation and species identification using average nucleotide identity (ANI) value

Sequencing reads were assembled using flye 2.9 with default options (*1*). The assembled sequences were evaluated for the average nucleotide identity using FastANI (*3*) with "–fragLen 1000" option against 404 assemblies of Mycobacterium comprising 214 identified spp., 13 sub-spp., and 177 not identified spp.

References

1. Kolmogorov M, Yuan J, Lin Y, Pevzner PA. Assembly of long, error-prone reads using repeat graphs. Nat Biotechnol. 2019;37:540–6. PubMed https://doi.org/10.1038/s41587-019-0072-8

2. Wick RR, Judd LM, Gorrie CL, Holt KE. Unicycler: resolving bacterial genome assemblies from short and long sequencing reads. PLOS Comput Biol. 2017;13:e1005595. PubMed https://doi.org/10.1371/journal.pcbi.1005595

3. Jain C, Rodriguez-R LM, Phillippy AM, Konstantinidis KT, Aluru S. High throughput ANI analysis of 90K prokaryotic genomes reveals clear species boundaries. Nat Commun. 2018;9:5114. PubMed

Appendix Table 1. Sampling dates for whole-genome sequencing of *Mycobacterium novusgordonae* and *M. shingordonae* strains

Isolate	Sampling dates
MN1 (TY813)	20210330
MN2	20221025
MS1 (TY814)	20201014, 20210707, 20220316
MS2 (TY815)	20220208, 20220325
MS3	20220830

Appendix Table 2. Reference sequence list of average nucleotides identifying mycobacteria species

Scientific name	NCBI RefSeq accession no.
Mycobacterium ahvazicum	GCF_022370835.1
Mycobacterium alsense	GCF_016592155.1
Mycobacterium angelicum	GCF_000015305.1
Mycobacterium aquaticum	GCF_002102265.1
Mycobacterium arosiense	GCF_010731755.1
Mycobacterium asiaticum	GCF_000382405.1
Mycobacterium attenuatum	GCF_002356495.1
Mycobacterium avium	GCF_010731935.1
Mycobacterium avium subsp. *avium*	GCF_000373905.1
Mycobacterium avium subsp. *hominissuis*	GCF_002982225.1
Mycobacterium avium subsp. *paratuberculosis*	GCF_000262165.1
Mycobacterium basiliense	GCF_000364405.1

Scientific name	NCBI RefSeq accession no.
Mycobacterium bohemicum	GCF_012223425.1
Mycobacterium botniense	GCF_900141695.1
Mycobacterium bouchedurhonense	GCF_000419295.1
Mycobacterium bourgelatii	GCF_002148195.1
Mycobacterium branderi	GCF_015689175.1
Mycobacterium canettii	GCF_010731795.1
Mycobacterium celatum	GCF_002102185.1
Mycobacterium colombiense	GCF_010728155.1
Mycobacterium conspicuum	GCF_010723575.1
Mycobacterium cookii	GCF_010731735.1
Mycobacterium crocinum	GCF_010731815.2
Mycobacterium decipiens	GCF_002086455.1
Mycobacterium diernhoferi	GCF_000353205.1
Mycobacterium dioxanotrophicus	GCF_000709305.1
Mycobacterium doricum	GCF_000523635.1
Mycobacterium europaeum	GCF_000523975.1
Mycobacterium florentinum	GCF_010728525.1
Mycobacterium fragae	GCF_022370755.1
Mycobacterium frederiksbergense	GCF_000455205.1
Mycobacterium gastri	GCF_000455125.1
Mycobacterium genavense	GCF_000620625.1
Mycobacterium goodii	GCF_010723415.1
Mycobacterium gordonae	GCF_022374895.1
Mycobacterium grossiae	GCF_002104675.1
Mycobacterium hemophilum	GCF_010731855.1
Mycobacterium heckeshornense	GCF_022179545.1
Mycobacterium heidelbergense	GCF_001570465.1
Mycobacterium helveticum	GCF_010731295.1
Mycobacterium heraklionense	GCF_001245615.1
Mycobacterium hodleri	GCF_001570425.1
Mycobacterium holsaticum	GCF_900078665.2
Mycobacterium innocens	GCF_002086915.1
Mycobacterium interjectum	GCF_019645835.1
Mycobacterium intermedium	GCF_010731875.1

Scientific name	NCBI RefSeq accession no.
Mycobacterium intracellulare	GCF_010731895.1
Mycobacterium intracellulare subsp. *chimaera*	GCF_002101785.1
Mycobacterium intracellulare subsp. *intracellulare*	GCF_000972905.1
Mycobacterium intracellulare subsp. *yongonense*	GCF_000972935.1
Mycobacterium kansasii	GCF_001021385.1
Mycobacterium koreense	GCF_003112775.1
Mycobacterium kubicae	GCF_008329645.1
Mycobacterium kyogaense	GCF_001050035.1
Mycobacterium kyorinense	GCF_001050015.1
Mycobacterium lacus	GCF_002086715.1
Mycobacterium lehmannii	GCF_001414075.1
Mycobacterium lentiflavum	GCF_001414095.1
Mycobacterium leprae	GCF_017876775.1
Mycobacterium lepromatosis	GCF_001644575.1
Mycobacterium liflandii	GCF_010731535.1
Mycobacterium malmoense	GCF_013466425.1
Mycobacterium mantenii	GCF_001440005.1
Mycobacterium marinum	GCF_001440085.1
Mycobacterium marseillense	GCF_001440125.1
Mycobacterium montefiorense	GCF_001440105.1
Mycobacterium mungi	GCF_001440135.1
Mycobacterium nebraskense	GCF_001440155.1
Mycobacterium neglectum	GCF_001440185.1
Mycobacterium neumannii	GCF_001440205.1
Mycobacterium noviomagense	GCF_001440225.1
Mycobacterium novum	GCF_001440245.1
Mycobacterium numidiamassiliense	GCF_001440265.1
Mycobacterium orygis	GCF_001440275.1
Mycobacterium ostraviense	GCF_001440305.1
Mycobacterium palauense	GCF_001426545.1
Mycobacterium pallens	GCF_001428895.1
Mycobacterium palustre	GCF_002101935.1
Mycobacterium paraense	GCF_001545925.1
Mycobacterium paraffinicum	GCF_015708165.1

Scientific name	NCBI RefSeq accession no.
Mycobacterium paragordonae	GCF_902509475.1
Mycobacterium paraintracellulare	GCF_001307545.1
Mycobacterium parascrofulaceum	GCF_000277125.1
Mycobacterium paraseoulense	GCF_000157895.3
Mycobacterium paraterrae	GCF_003253775.1
Mycobacterium parmense	GCF_021696875.1
Mycobacterium persicum	GCF_001583415.1
Mycobacterium pseudokansasii	GCF_013349145.1
Mycobacterium pseudoshottsii	GCF_001499905.1
Mycobacterium rhizamassiliense	GCF_001499965.1
Mycobacterium riyadhense	GCF_000195955.2
Mycobacterium rufum	GCF_001632805.1
Mycobacterium saskatchewanense	GCF_010727945.1
Mycobacterium scrofulaceum	GCF_001722335.1
Mycobacterium senegalense	GCF_002102175.1
Mycobacterium senriense	GCF_002101675.1
Mycobacterium seoulense	GCF_019645855.1
Mycobacterium sherrisii	GCF_003609695.1
Mycobacterium shigaense	GCF_002101775.1
Mycobacterium shimoidei	GCF_002086735.1
Mycobacterium shinjukuense	GCF_010727605.1
Mycobacterium shottsii	GCF_002250655.1
Mycobacterium simiae	GCF_002116635.1
Mycobacterium simulans	GCF_010727125.1
Mycobacterium sp. *012931*	GCF_009936235.1
Mycobacterium sp. *1081908.1*	GCF_000613245.1
Mycobacterium sp. *1100029.7*	GCF_001049355.1
Mycobacterium sp. *1164966.3*	GCF_010727725.1
Mycobacterium sp. *1165178.9*	GCF_900078685.2
Mycobacterium sp. *1165196.3*	GCF_010726955.1
Mycobacterium sp. *1245111.1*	GCF_010728925.1
Mycobacterium sp. *1245499.0*	GCF_005670605.1
Mycobacterium sp. *1245801.1*	GCF_022374935.1
Mycobacterium sp. *1245805.9*	GCF_019645875.1

Scientific name	NCBI RefSeq accession no.
Mycobacterium sp. *1245852.3*	GCF_000234585.1
Mycobacterium sp. *1274761.0*	GCF_002102395.1
Mycobacterium sp. *141*	GCF_010726245.1
Mycobacterium sp. *1423905.2*	GCF_001044255.1
Mycobacterium sp. *1465703.0*	GCF_019456655.1
Mycobacterium sp. *1482292.6*	GCF_000723385.1
Mycobacterium sp. *1554424.7*	GCF_000184435.1
Mycobacterium sp. *155*	GCF_002982215.1
Mycobacterium sp. *20KCMC460*	GCF_001044245.1
Mycobacterium sp. *3519A*	GCF_022374915.1
Mycobacterium sp. *4858*	GCF_001552715.1
Mycobacterium sp. *852002–10029_SCH5224772*	GCF_010722995.1
Mycobacterium sp. *852002–30065_SCH5024008*	GCF_019075575.1
Mycobacterium sp. *852002–40037_SCH5390672*	GCF_001673535.1
Mycobacterium sp. *852002–50816_SCH5313054-b*	GCF_001665235.1
Mycobacterium sp. *852002–51057_SCH5723018*	GCF_001673555.1
Mycobacterium sp. *852002–51152_SCH6134967*	GCF_001673415.1
Mycobacterium sp. *852002–51163_SCH5372311*	GCF_001673405.1
Mycobacterium sp. *852002–51613_SCH5001154*	GCF_001673615.1
Mycobacterium sp. *852002–51971_SCH5477799-a*	GCF_001673055.1
Mycobacterium sp. *852002–53434_SCH5985345*	GCF_001668615.1
Mycobacterium sp. *852013–51886_SCH5428379*	GCF_001672975.1
Mycobacterium sp. *852014–50255_SCH5639931*	GCF_001672995.1
Mycobacterium sp. *852014–52144_SCH5372336*	GCF_001665295.1
Mycobacterium sp. *852014–52450_SCH5900713*	GCF_001667315.1
Mycobacterium sp. *ACS1612*	GCF_001665645.1
Mycobacterium sp. *ACS4054*	GCF_001665875.1
Mycobacterium sp. *AT1*	GCF_001667185.1
Mycobacterium sp. *AZCC_0083*	GCF_001667035.1
Mycobacterium sp. *CnD-18–1*	GCF_001667105.1
Mycobacterium sp. *DBP42*	GCF_001667115.1
Mycobacterium sp. *DL440*	GCF_001667425.1
Mycobacterium sp. *DL592*	GCF_001667145.1
Mycobacterium sp. *E1214*	GCF_001667155.1

Scientific name	NCBI RefSeq accession no.
Mycobacterium sp. *E1319*	GCF_001665575.1
Mycobacterium sp. *E1386*	GCF_001665605.1
Mycobacterium sp. *E1715*	GCF_001665615.1
Mycobacterium sp. *E1747*	GCF_001667075.1
Mycobacterium sp. *E183*	GCF_001667505.1
Mycobacterium sp. *E188*	GCF_001667275.1
Mycobacterium sp. *E2238*	GCF_001666755.1
Mycobacterium sp. *E2327*	GCF_001667745.1
Mycobacterium sp. *E2462*	GCF_001672745.1
Mycobacterium sp. *E2479*	GCF_001667775.1
Mycobacterium sp. *E2497*	GCF_001667735.1
Mycobacterium sp. *E2699*	GCF_001666935.1
Mycobacterium sp. *E2733*	GCF_001672665.1
Mycobacterium sp. *E2989*	GCF_001672675.1
Mycobacterium sp. *E3198*	GCF_001672685.1
Mycobacterium sp. *E3247*	GCF_001667925.1
Mycobacterium sp. *E3251*	GCF_001668695.1
Mycobacterium sp. *E3298*	GCF_001666745.1
Mycobacterium sp. *E3305*	GCF_001667585.1
Mycobacterium sp. *E3339*	GCF_001667595.1
Mycobacterium sp. *E342*	GCF_001667015.1
Mycobacterium sp. *E735*	GCF_001666835.1
Mycobacterium sp. *E740*	GCF_001666785.1
Mycobacterium sp. *E787*	GCF_001666915.1
Mycobacterium sp. *E796*	GCF_001667665.1
Mycobacterium sp. *E802*	GCF_001666955.1
Mycobacterium sp. *EPa45*	GCF_001672895.1
Mycobacterium sp. *GA-1285*	GCF_001668725.1
Mycobacterium sp. *GA-1841*	GCF_001672815.1
Mycobacterium sp. *GA-2829*	GCF_001666875.1
Mycobacterium sp. *H4Y*	GCF_001954135.1
Mycobacterium sp. *IDR2000157661*	GCF_001954195.1
Mycobacterium sp. *IEC1808*	GCF_001954215.1
Mycobacterium sp. *IS-1264*	GCF_001954275.1

Scientific name	NCBI RefSeq accession no.
Mycobacterium sp. *IS-2888*	GCF_001953975.1
Mycobacterium sp. *IS-836*	GCF_001954045.1
Mycobacterium sp. *ITM-2016–00316*	GCF_900157385.1
Mycobacterium sp. *ITM-2016–00318*	GCF_900157375.1
Mycobacterium sp. *IWGMT90018–18076*	GCF_900157365.1
Mycobacterium sp. *JS623*	GCF_001652545.1
Mycobacterium sp. *M1*	GCF_010730575.1
Mycobacterium sp. *MAC_011194_8550*	GCF_001673155.1
Mycobacterium sp. *MAC_080597_8934*	GCF_001669335.1
Mycobacterium sp. *MAG1*	GCF_001665395.1
Mycobacterium sp. *Marseille-P9652*	GCF_001672915.1
Mycobacterium sp. *MFM001*	GCF_001667625.1
Mycobacterium sp. *MOTT36Y*	GCF_001667785.1
Mycobacterium sp. *MS1601*	GCF_001666895.1
Mycobacterium sp. *MYCO198283*	GCF_001854525.1
Mycobacterium sp. *NAZ190054*	GCF_002838065.1
Mycobacterium sp. *NS-7484*	GCF_002101585.1
Mycobacterium sp. *OAS707*	GCF_009729375.1
Mycobacterium spongiae	GCF_001942625.1
Mycobacterium sp. *P7213*	GCF_009729415.1
Mycobacterium sp. *PS03–16*	GCF_001886515.1
Mycobacterium sp. *PSTR-4-N*	GCF_002086485.1
Mycobacterium sp. *PYR15*	GCF_001984215.1
Mycobacterium sp. *QGD 101*	GCF_002982275.1
Mycobacterium sp. *Root135*	GCF_002043095.1
Mycobacterium sp. *Root265*	GCF_000497265.2
Mycobacterium sp. *shizuoka-1*	GCF_900176255.2
Mycobacterium sp. *SM1*	GCF_009729395.1
Mycobacterium sp. *SP-6446*	GCF_008329605.1
Mycobacterium sp. *'sulfur cave'*	GCF_008329585.1
Mycobacterium sp. *TKK-01–0059*	GCF_008329535.1
Mycobacterium sp. *UM_11*	GCF_002592005.1
Mycobacterium sp. *UM_3*	GCF_002723835.1
Mycobacterium sp. *UM_CSW*	GCF_002245535.1

Scientific name	NCBI RefSeq accession no.
Mycobacterium sp. *UM_Kg27*	GCF_002245615.1
Mycobacterium sp. *UM_NZ2*	GCF_003402475.1
Mycobacterium sp. *UM_WGJ*	GCF_002335685.1
Mycobacterium sp. *URHB0044*	GCF_900240945.1
Mycobacterium sp. *WUMAC-025*	GCF_900240975.1
Mycobacterium sp. *WUMAC-067*	GCF_900292015.1
Mycobacterium sp. *WY10*	GCF_002968285.1
Mycobacterium sp. *Y57*	GCF_002968335.1
Mycobacterium sp. *YC-RL4*	GCF_010729665.1
Mycobacterium stomatepiae	GCF_000328565.1
Mycobacterium syngnathidarum	GCF_003408705.1
Mycobacterium szulgai	GCF_010729305.1
Mycobacterium talmoniae	GCF_003851485.1
Mycobacterium terramassiliense	GCF_002101875.1
Mycobacterium timonense	GCF_003254575.1
Mycobacterium triplex	GCF_002219285.1
Mycobacterium tuberculosis	GCF_003284965.1
Mycobacterium tuberculosis subsp. *tuberculosis*	GCF_002101555.1
Mycobacterium tuberculosis variant africanum	GCF_900566075.1
Mycobacterium tuberculosis variant bovis BCG	GCF_900566055.1
Mycobacterium tuberculosis variant bovis	GCF_900566065.1
Mycobacterium tuberculosis variant caprae	GCF_003719305.1
Mycobacterium tuberculosis variant microti	GCF_000164135.1
Mycobacterium tuberculosis variant pinnipedii	GCF_002591975.1
Mycobacterium uberis	GCF_002102355.1
Mycobacterium ulcerans	GCF_002102255.1
Mycobacterium ulcerans subsp. *shinshuense*	GCF_900604305.1
Mycobacterium vicinigordonae	GCF_003855255.1
Mycobacterium virginiense	GCF_010726505.1
Mycobacterium vulneris	GCF_004745805.1
Mycobacterium xenopi	GCF_005924235.1
Mycobacteroides abscessus	GCF_004570325.1
Mycobacteroides abscessus subsp. *abscessus*	GCF_005222675.1
Mycobacteroides abscessus subsp. *bolletii*	GCF_005938675.2

Scientific name	NCBI RefSeq accession no.
Mycobacteroides abscessus subsp. *massiliense*	GCF_011758805.1
Mycobacteroides chelonae	GCF_000613185.1
Mycobacteroides franklinii	GCF_014195125.1
Mycobacteroides immunogenum	GCF_014195195.1
Mycobacteroides salmoniphilum	GCF_014195025.1
Mycobacteroides saopaulense	GCF_007096635.1
Mycobacteroides sp. *CBMA 271*	GCF_007714185.1
Mycobacteroides sp. *CBMA 326*	GCF_009729295.1
Mycobacteroides sp. *H001*	GCF_009729335.1
Mycobacteroides sp. *H002*	GCF_009729275.1
Mycobacteroides sp. *H003*	GCF_009729315.1
Mycobacteroides sp. *H054*	GCF_009729305.1
Mycobacteroides sp. *H063*	GCF_009729185.1
Mycobacteroides sp. *H070*	GCF_009729215.1
Mycobacteroides sp. *H072*	GCF_009729205.1
Mycobacteroides sp. *H079*	GCF_009729195.1
Mycobacteroides sp. *H092*	GCF_009729175.1
Mycobacteroides sp. *H101*	GCF_009729075.1
Mycobacteroides sp. *H110*	GCF_009729095.1
Mycobacteroides sp. *HXVII*	GCF_009729085.1
Mycobacteroides sp. *HXXIII*	GCF_009729105.1
Mycobacteroides sp. *LB1*	GCF_000026445.2
Mycolicibacillus parakoreensis	GCF_902652685.1
Mycolicibacillus trivialis	GCF_003584745.1
Mycolicibacter algericus	GCF_014873705.1
Mycolicibacter arupensis	GCF_011745145.1
Mycolicibacter engbaekii	GCF_011694515.1
Mycolicibacter hiberniae	GCF_014202335.1
Mycolicibacter icosiumassiliensis	GCF_015710575.1
Mycolicibacterium agri	GCF_013404075.1
Mycolicibacterium aichiense	GCF_021183725.1
Mycolicibacterium alvei	GCF_013449725.1
Mycolicibacterium anyangense	GCF_018326145.1
Mycolicibacterium arabiense	GCF_018326285.1

Scientific name	NCBI RefSeq accession no.
Mycolicibacterium aromaticivorans	GCF_016313185.1
Mycolicibacterium aubagnense	GCF_019668465.1
Mycolicibacterium aurum	GCF_019710635.1
Mycolicibacterium austroafricanum	GCF_020217485.1
Mycolicibacterium bacteremicum	GCF_020217805.1
Mycolicibacterium baixiangningiae	GCF_000974705.1
Mycolicibacterium boenickei	GCF_010729895.1
Mycolicibacterium brisbanense	GCF_000878195.1
Mycolicibacterium brumae	GCF_017352375.1
Mycolicibacterium canariasense	GCF_017948465.1
Mycolicibacterium celeriflavum	GCF_018361265.1
Mycolicibacterium chitae	GCF_018455725.1
Mycolicibacterium chlorophenolicum	GCF_910591625.1
Mycolicibacterium chubuense	GCF_002086275.1
Mycolicibacterium conceptionense	GCF_019148985.1
Mycolicibacterium confluentis	GCF_020172665.1
Mycolicibacterium cosmeticum	GCF_020172685.1
Mycolicibacterium duvalii	GCF_022317005.1
Mycolicibacterium elephantis	GCF_021654635.1
Mycolicibacterium fallax	GCF_020181615.1
Mycolicibacterium farcinogenes	GCF_020181635.1
Mycolicibacterium flavescens	GCF_020181595.1
Mycolicibacterium fluoranthenivorans	GCF_021213805.1
Mycolicibacterium fortuitum	GCF_021916785.1
Mycolicibacterium fortuitum subsp. *acetamidolyticum*	GCF_022179425.1
Mycolicibacterium fortuitum subsp. *fortuitum*	GCF_022557175.1
Mycolicibacterium gadium	GCF_021648845.1
Mycolicibacterium gilvum	GCF_022348025.1
Mycolicibacterium hassiacum	GCF_022348085.1
Mycolicibacterium helvum	GCF_022348005.1
Mycolicibacterium hippocampi	GCF_010730355.1
Mycolicibacterium houstonense	GCF_023015925.1
*Mycolicibacterium in*subricum	GCF_000340435.2
Mycolicibacterium iranicum	GCF_900417275.1

Scientific name	NCBI RefSeq accession no.
Mycolicibacterium komanii	GCF_010729485.1
Mycolicibacterium lacusdiani	GCF_022374875.1
Mycolicibacterium litorale	GCF_000559085.1
Mycolicibacterium llatzerense	GCF_001792615.1
Mycolicibacterium lutetiense	GCF_010731115.1
Mycolicibacterium madagascariense	GCF_010730955.1
Mycolicibacterium mageritense	GCF_002086635.1
Mycolicibacterium malmesburyense	GCF_015482805.1
Mycolicibacterium mengxianglii	GCF_002982335.1
Mycolicibacterium monacense	GCF_900078675.2
Mycolicibacterium moriokaense	GCF_000222105.3
Mycolicibacterium mucogenicum	GCF_002086515.1
Mycolicibacterium murale	GCF_002086285.1
Mycolicibacterium neoaurum	GCF_021183785.1
Mycolicibacterium neworleansense	GCF_000069185.1
Mycolicibacterium nivoides	GCF_010722915.1
Mycolicibacterium novocastrense	GCF_000526915.1
Mycolicibacterium obuense	GCF_010725725.1
Mycolicibacterium parafortuitum	GCF_002086695.1
Mycolicibacterium peregrinum	GCF_019456675.1
Mycolicibacterium phlei	GCF_900108565.1
Mycolicibacterium phocaicum	GCF_001552315.1
Mycolicibacterium poriferae	GCF_010731595.1
Mycolicibacterium psychrotolerans	GCF_002101885.1
Mycolicibacterium pulveris	GCF_022370635.1
Mycolicibacterium rhodesiae	GCF_000612725.1
Mycolicibacterium rutilum	GCF_010726645.1
Mycolicibacterium sarraceniae	GCF_010726085.1
Mycolicibacterium sediminis	GCF_010725485.1
Mycolicibacterium septicum	GCF_010728325.1
Mycolicibacterium setense	GCF_010725885.1
Mycolicibacterium smegmatis	GCF_010730055.1
Mycolicibacterium sp. *018_SC-01_001*	GCF_004924335.1
Mycolicibacterium sp. *BK556*	GCF_002086125.1

Scientific name	NCBI RefSeq accession no.
Mycolicibacterium sp. *BK607*	GCF_000805385.1
Mycolicibacterium sp. *BK634*	GCF_002102345.1
Mycolicibacterium sp. *CBMA 213*	GCF_010728725.1
Mycolicibacterium sp. *CBMA 226*	GCF_000696715.1
Mycolicibacterium sp. *CBMA 230*	GCF_010730195.1
Mycolicibacterium sp. *CBMA 234*	GCF_021183845.1
Mycolicibacterium sp. *CBMA 247*	GCF_010731615.1
Mycolicibacterium sp. *CBMA 293*	GCF_002102065.1
Mycolicibacterium sp. *CBMA 294*	GCF_010731635.1
Mycolicibacterium sp. *CBMA 295*	GCF_000379865.1
Mycolicibacterium sp. *CBMA 311*	GCF_002086155.1
Mycolicibacterium sp. *CBMA 329*	GCF_010731715.1
Mycolicibacterium sp. *CBMA 331*	GCF_002102415.1
Mycolicibacterium sp. *CBMA 334*	GCF_000966355.1
Mycolicibacterium sp. *CBMA 335*	GCF_002157835.1
Mycolicibacterium sp. *CBMA 360*	GCF_002101845.1
Mycolicibacterium sp. *CBMA 361*	GCF_002101735.1
Mycolicibacterium sp. *CH28*	GCF_006864685.1
Mycolicibacterium sp. *CR10*	GCF_001021505.1
Mycolicibacterium sp. *F2034L*	GCF_010730745.1
Mycolicibacterium sp. *GESEQ-9*	GCF_001907675.1
Mycolicibacterium sp. *GF69*	GCF_010727475.1
Mycolicibacterium sphagni	GCF_002104765.1
Mycolicibacterium sp. *NCC-Tsukiji*	GCF_010731775.1
Mycolicibacterium sp. *OfavD-34-C*	GCF_002086115.1
Mycolicibacterium sp. *P1–18*	GCF_002102025.1
Mycolicibacterium sp. *P9–22*	GCF_005670685.2
Mycolicibacterium sp. *P9–64*	GCF_022430545.1
Mycolicibacterium sp. *PAM1*	GCF_010731655.1
Mycolicibacterium sp. *S2–37*	GCF_002101965.1
Mycolicibacterium sp. *TY66*	GCF_001499835.1
Mycolicibacterium sp. *TY81*	GCF_900232995.1
Mycolicibacterium sp. *YH-1*	GCF_010723735.1
Mycolicibacterium stellerae	GCF_010727325.1

Scientific name	NCBI RefSeq accession no.
Mycolicibacterium thermoresistibile	GCF_002086165.1
Mycolicibacterium tokaiense	GCF_010731675.1
Mycolicibacterium tusciae	GCF_002086775.1
Mycolicibacterium vaccae	GCF_002356315.1
Mycolicibacterium vanbaalenii	GCF_010731695.1
Mycolicibacterium vinylchloridicum	GCF_002086795.1
Mycolicibacterium wolinskyi	GCF_002102155.1
Mycolicibacter kumamotonensis	GCF_002101815.1
Mycolicibacter longobardus	GCF_000253375.1
Mycolicibacter minnesotensis	GCF_004014805.1
Mycolicibacter nonchromogenicus	GCF_001605725.1
Mycolicibacter senuensis	GCF_010723305.1
Mycolicibacter sinensis	GCF_010731575.1
Mycolicibacter sp. *MYC017*	GCF_004014795.1
Mycolicibacter sp. *MYC098*	GCF_000214155.1
Mycolicibacter sp. *MYC101*	GCF_018278905.1
Mycolicibacter sp. *MYC123*	GCF_002101705.1
Mycolicibacter sp. *MYC340*	GCF_004355025.1
Mycolicibacter terrae	GCF_012396425.1

Appendix Table 3. Antimicrobial drug susceptibility data for the new *Mycobacterium gordonae*-clade species

Drug	MN1	MN2	MS1	MS2	MS3
Clarithromycin	0.5*	0.5	>32	1	0.5
Streptomycin	4	8	8	8	4
Kanamycin	4	4	16	8	8
Amikacin	2	4	8	8	8
Ethambutol	1	0.5	0.5	0.5	2
Rifampin	0.5	0.5	1	0.5	1
Levofloxacin	0.5	2	1	1	2
Ethionamide	16	16	16	16	16

*MIC; µg/mL

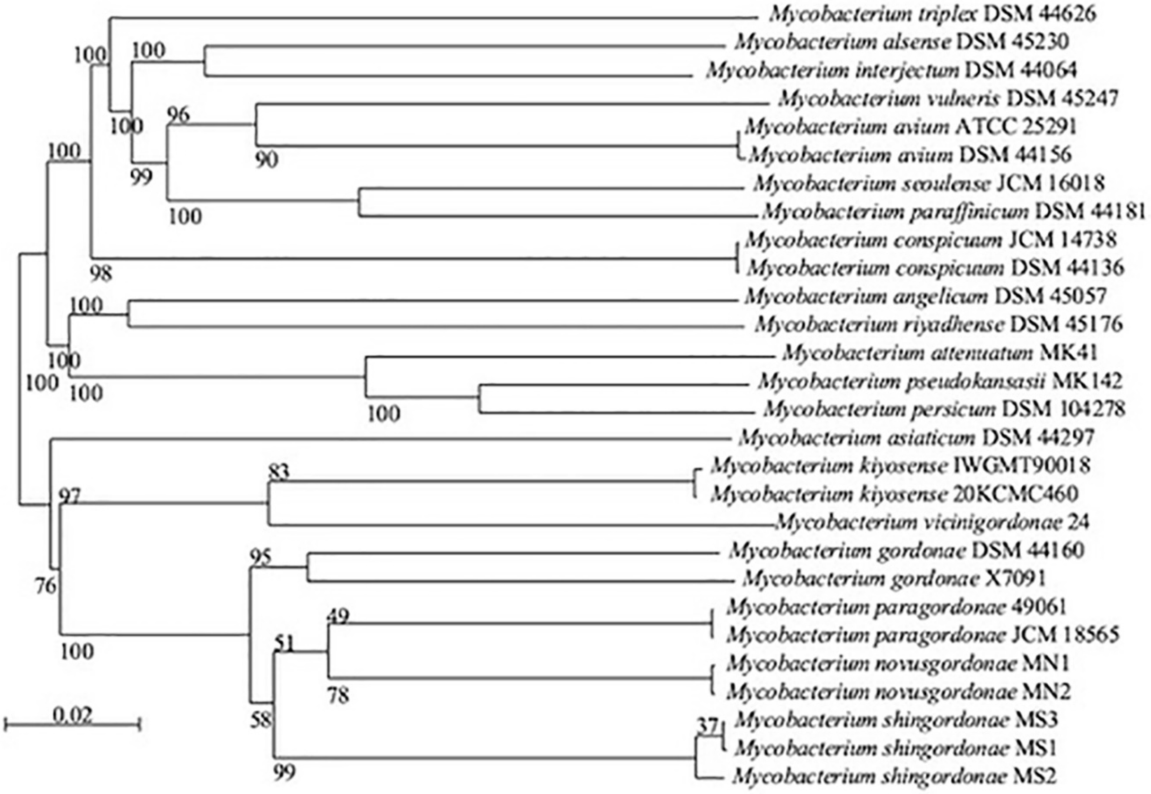

Appendix Figure 1. Whole-genome sequence based phylogenetic tree generated with TYGS. Tree inferred with FastME 2.1.6.1 from GBDP distances calculated from genome sequences. The branch lengths are scaled in terms of GBDP distance formula d5. The numbers above branches are GBDP pseudo-bootstrap support values >60% from 100 replications, with an average branch support of 91.8%, demonstrating the robustness of the inferred relationships. The tree was rooted at the midpoint.

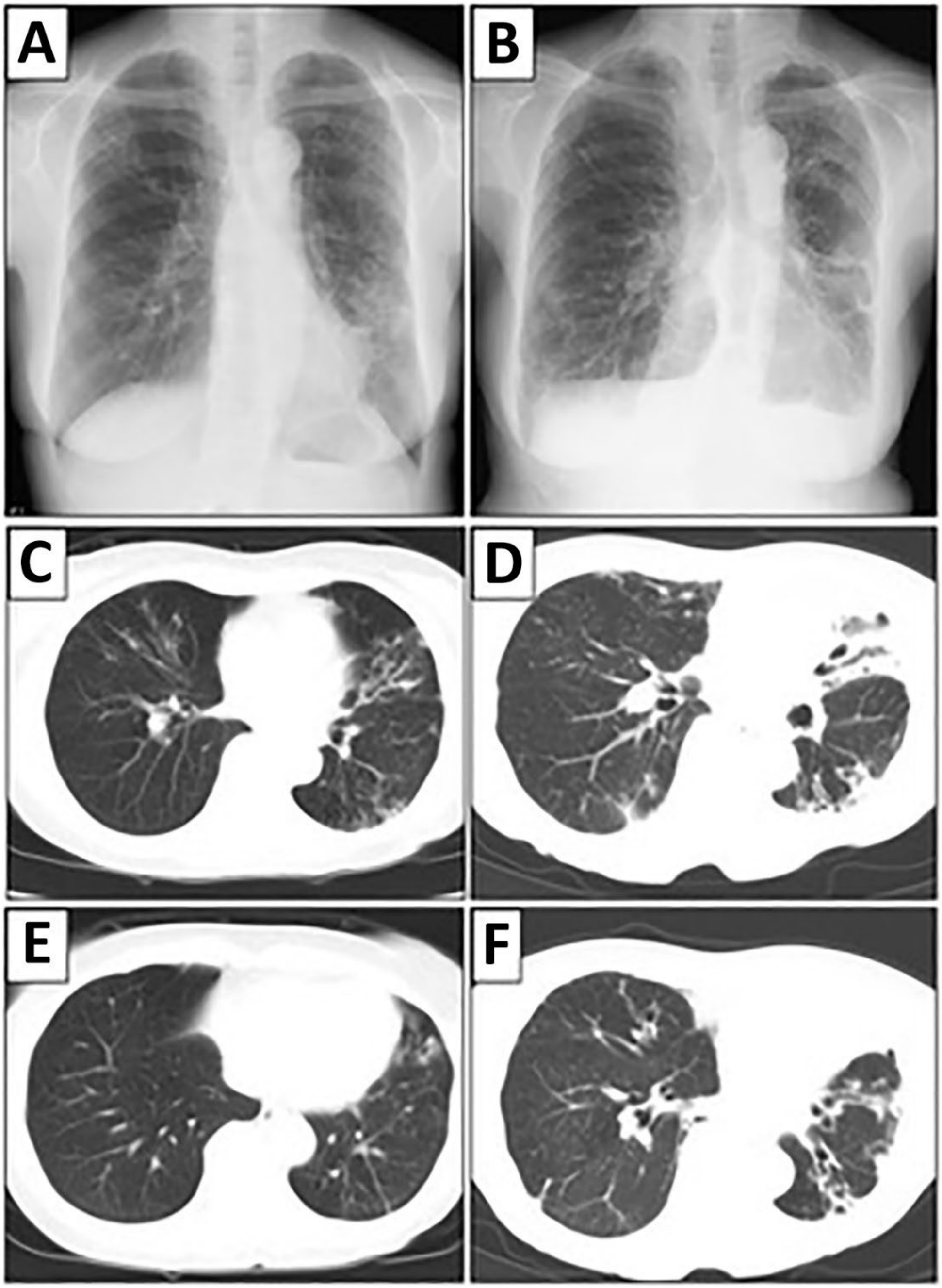

Appendix Figure 2. Chest x-ray (A, B) and computed tomography (C, D, E, F), at age 68 (A, C, E), and at age 80 (B, D, F) are presented. The images revealed progressive worsening of centrilobular nodules and bronchiectasis in the middle and bilateral lower lobes.
